# Meckel's Diverticulum: The “Great Mimic” but Often a Forgotten Cause of Acute Abdomen during Pregnancy

**DOI:** 10.1155/2022/2383075

**Published:** 2022-06-07

**Authors:** Beatriz Féria, Madalena Trindade, Maria João Palma, Joana Figueiredo, Filipa Passos

**Affiliations:** ^1^Obstetrics and Gynecology, Hospital Garcia de Orta, Almada, Portugal; ^2^General Surgery, Hospital Garcia de Orta, Almada, Portugal

## Abstract

Meckel's diverticulitis is an extremely rare event during pregnancy. Its diagnosis is often difficult and can result in higher maternal and fetal morbimortality. We describe a case of a 40-year-old healthy pregnant woman at 33 weeks of gestation who presented with abdominal pain and tender abdomen, leukocytosis, and elevated PCR. The imagiological exams were not conclusive. After an urgent caesarean section due to worsening of clinical status and nonreassuring fetal well-being, a laparotomy revealed a distended, necrotic, and perforated Meckel's diverticulum.

## 1. Introduction

Acute abdomen during pregnancy is a rare condition, with a reported incidence of one in 500-635 patients [1, 2]. Perforation of Meckel's diverticulum is an exceptional but potentially life-threatening condition during pregnancy. When assessing a pregnant patient with an acute abdomen, its diagnosis must be considered and early management is imperative to avoid complications. We report a rare case of an acute abdomen caused by infection and perforation of Meckel's diverticulum in the third trimester of pregnancy.

## 2. Case Presentation

A 40-year-old woman, primigravid, presented at our emergency department at 33 weeks of gestation with a diffuse abdominal pain for two days, more intense at the left iliac fossa, sharp and not responsive to analgesia. The pain was associated with nausea, vomiting, and constipation for a day. She had no previous medical, gynecological, or surgical history, and her antenatal course had been uneventful.

On physical examination, the patient had a blood pressure of 117/67 mmHg and pulse rate of 93 bpm and was apyretic. The abdomen was distended and tender. There was no evidence of uterine contractions or fetal distress. The blood work revealed elevated white blood cell count (21,300/mL with 88% neutrophils) and elevated CRP (19,1 mg/dL). Abdominal ultrasound was normal so it was decided to perform an abdominal-pelvic computerized tomography scan (CT scan) that showed densification of pericolic fat tissue suggesting an inflammatory process of indeterminate nature and no other alterations.

Corticosteroids for fetal lung maturation and empirical broad-spectrum antibiotics were initiated with temporarily clinical improvement. Despite that, on the following day, the patient presented intense pain, abdominal guarding, and rebound as well as an increase in inflammatory parameters. A second abdominal ultrasound was performed but was nonconclusive.

Differential diagnosis included appendicitis, bowel obstruction, perforated ulcer, inflammatory bowel disease, and diverticulitis. Obstetrical and gynecological causes were not excluded but were less plausible.

**Figure 1:**
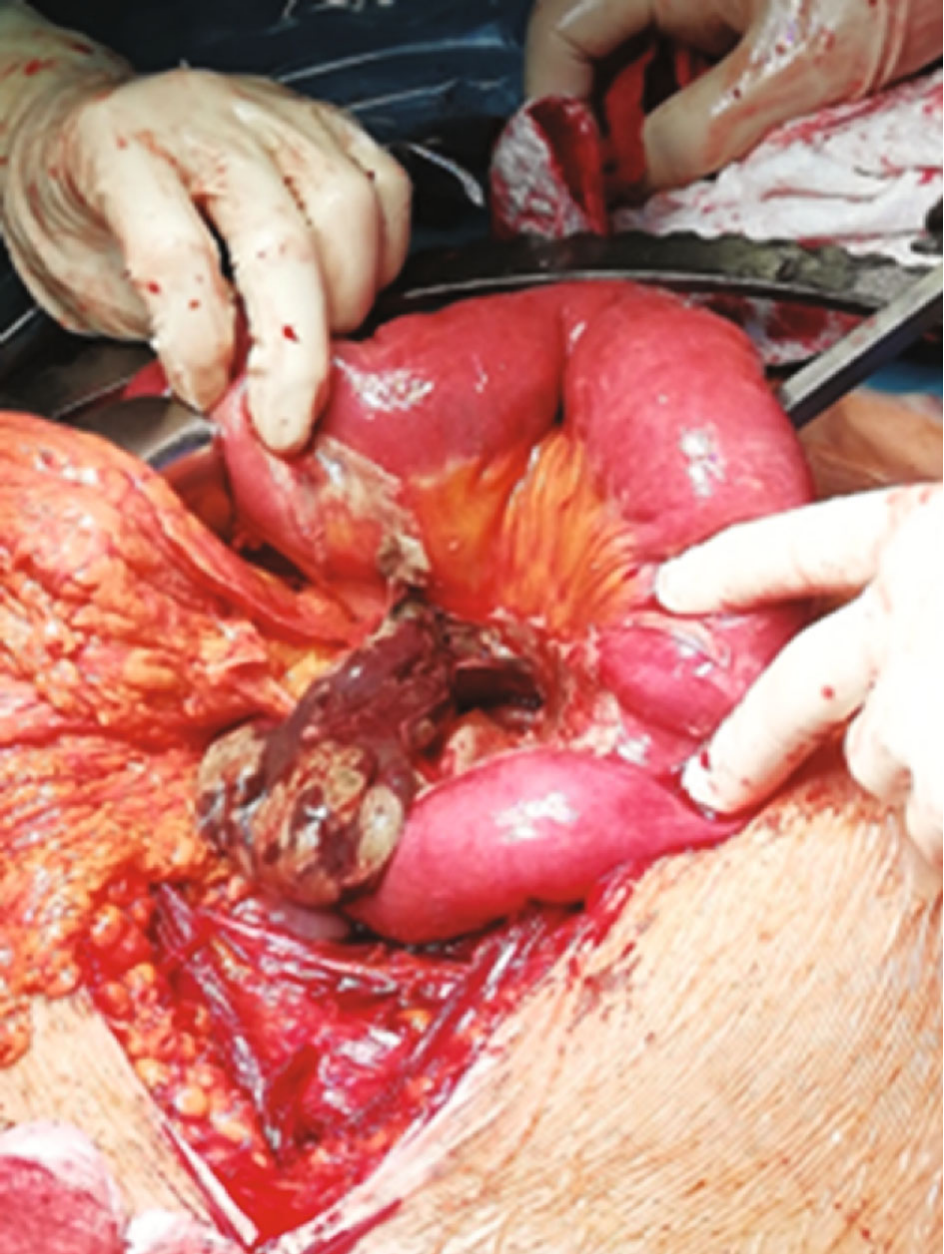
Distended and necrotic Meckel's diverticulum.

**Figure 2:**
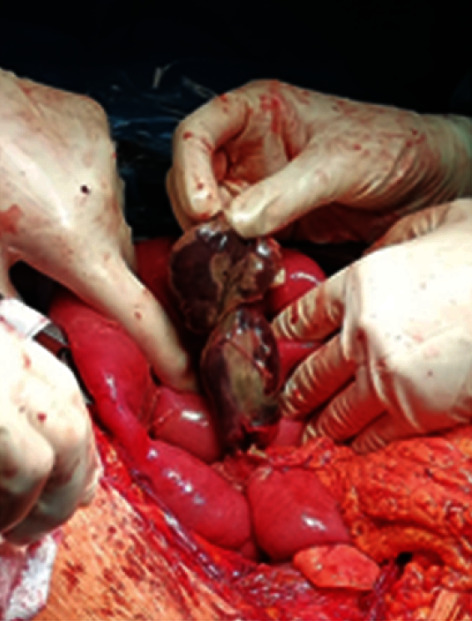
Perforation site of Meckel's diverticulum.

**Figure 3:**
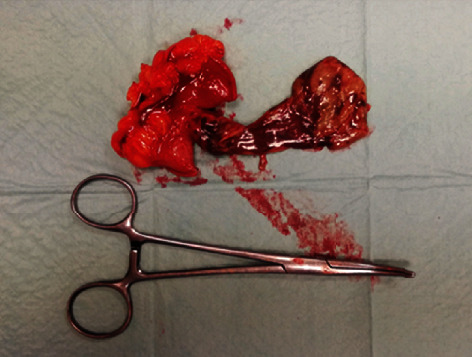
Meckel's diverticulum true dimensions (9 cm in length and 3 cm in diameter).

Facing an acute abdomen and also a pathological fetal cardiotocography, an urgent caesarean section was performed followed by a laparotomy. A male infant was born weighing 1966 grams with Apgar score of 9 and 10 at one and five minutes. It was identified purulent discharge on the abdominal cavity as well as a distended and necrotic Meckel's diverticulum with 9 cm in length and 3 cm in diameter, with a perforation site (Figures 1, 2, and 3). Segmental small bowel resection with mechanical anastomosis was performed.

The histological report confirmed the diagnosis and reported no ectopic mucosa nor malignancy. The patient had an eventful recovery and was discharged after four days.

## 3. Discussion

Meckel's diverticulum (MD) is a true diverticulum containing all layers of the intestinal wall. It is the most common congenital anomaly of the gastrointestinal track and results from the failure of the omphalomesenteric duct to involute during the 5th to 7th weeks of gestation. The reported prevalence in general population is approximately 2%. Symptomatic Meckel's diverticulum during pregnancy is an extremely rare event. There are only 38 cases reported so far since 1949 [3–6] and 21 cases describing perforation of Meckel's diverticulum [5, 6].

This condition has been classically described as the “rule of twos”: prevalence of 2% in population, twice as common in men than women, located 2 feet from the ileocecal valve, and can be 2 inches wide and long [7]. Typically, MD is clinically silent and only 2-4% of people become symptomatic, usually in children [7, 8], with the risk decreasing with age.

The most common complications are obstruction, intussusception, inflammation or diverticulitis, hemorrhage, and perforation. Obstruction is more frequent amongst adults, whereas bleeding is most commonly observed in children due to ulceration caused by acid secretion by ectopic gastric mucosa [9]. Rarely, fistulae and malignancy can occur with carcinoid tumor being the most frequent [6, 7]. MD can be discovered incidentally during abdominal exploration or diagnostic imaging.

The diagnosis of MD during pregnancy can be challenging as it often presents with unspecific symptoms such as abdominal pain, nausea, vomiting, and obstipation which overlap with other conditions and can also occur physiologically in pregnancy.

Additionally, anatomical and physiologic changes like gravid uterus changes, mild to moderate leukocytosis, and tachycardia are considered normal, making the evaluation even more difficult. The main differential diagnosis is with appendicitis as it is the first nonobstetric cause of acute abdomen in pregnancy with an incidence of 0.04%-0.2% [10] and both can cause pain on the right iliac fossa. MD complication should be suspected specially if imaging studies reveal a healthy appendix. Other causes of acute abdomen include cholecystitis (1/500 to 1/3000 [11]), pancreatitis (1/10 000 [12]), bowel obstruction, and urolithiasis, amongst others. In pregnancy, symptomatic MD may mimic obstetric conditions like preterm labor, chorioamnionitis, or placental abruption [5]. Ectopic pregnancy is a diagnosis that should be considered at the beginning of the pregnancy as it is an important cause of acute abdomen and its presentation can be catastrophic [13].

Ultrasound is often the first and most used diagnostic modality [6]. It is fast, noninvasive and involves no radiation; however, its sensibility is very limited as it is operator dependent and has limited field of view due to pregnant uterus. Computer tomography (CT) is usually the gold standard for diagnosis of several abdominal pathologies and can be utilized in pregnant women. Nevertheless, in a recent systematic review, CT had a very low diagnostic value (14.7%) [6] and exposes the fetus to radiation. MRI without gadolinium, if available, is a good alternative to CT because of its higher sensitivity and no radiation exposure [6, 14]. Technetium-99m (99mTc) pertechnetate, also known as a Meckel's scan, is a modality used to diagnose MD with good results, particularly in children. Although there are no reports of its use in this context, it is considered safe and might be a potential tool when suspecting MD. Overall, the role of imaging studies remains limited and the diagnosis remains one of clinical suspicion [5].

Management of MD usually involves an exploratory laparoscopy or laparotomy with surgical resection: either diverticulectomy or segmental bowel resection in case of ectopic tissue at the diverticular base, intestinal ischemia, or perforation at the diverticular base [14]. Laparoscopy has been performed in early pregnancy with no adverse fetal outcome [6]. A laparotomy may be more suitable in the third trimester given the larger uterus and the possible need of delivery. Although there is a report of one case managed with antibiotics and surgery performed only four months postpartum [15], conservative management failed in all other cases; therefore, it is not advisable because of the poor outcomes [6]. The optimal management of the incidentally discovered MD is not consensual; some authors advocate that given the high-risk complications and difficult diagnosis during pregnancy, prophylactic removal is reasonable [9], while others do not support it, arguing that leaving an incidentally detected MD in situ reduces the risk of postoperative complications without increasing late complications [16].

Regarding pregnancy, the rate of MD complication does not seem higher than in general population but the question remains as to whether is there any mechanism during pregnancy that might help the occurrence of complications. Can the growing uterus compress the bowel facilitating an obstruction/loop process? On the other hand, there seems to be a higher rate of perforation of MD during pregnancy, between 40.7% and 57% [5, 6], whereas in general population, the rate is 7% [9]. This might be due to not only the difficult diagnosis but also the reluctance to perform invasive exams and procedures, resulting in a delayed diagnosis [5]. Perforation of MD has been present in all cases of maternal death registered so far, although all of these occurred before 1973 [5].

Obstetric outcomes are difficult to evaluate due to the low number of cases. It has been reported a maternal mortality of 16%, fetal mortality of 13%, and incidence of preterm delivery of 25% [5]. According to a recent systematic review, that includes all cases registered between 1990 and 2021, the majority of cases occurred on the second (48.1%) and third trimester (48.1%), and only 3.7% happened on the first trimester [6]. In all 27 cases, the right preoperative diagnosis was correct in only 2 cases (7.4%). There were 10 cases of preterm delivery, 1 spontaneous late abortion at 20 weeks of gestation in a patient that presented with massive gastrointestinal bleeding, and 1 stillbirth at 24 weeks of gestation in a patient with a perforated MD; both of these could be attributable to delayed presentation or diagnosis. There were no cases of maternal death or other morbidities except for paralytic ileus [6].

Our case demonstrates the diagnostic challenge of an acute abdomen in pregnancy and the serious consequences that Meckel's diverticulum can have in pregnancy. The initial patient's evaluation was not easy considering a third trimester pregnancy and large uterus. The fact that the pain was more intense at the left abdominal quadrant was a confounding factor as well as the initial clinical improvement with antibiotics and analgesia. At first, the most likely diagnosis did not include MD but rather appendicitis and diverticulitis. Despite the general reluctance of performing radiation exams, we decided to do a CT scan at an early stage. Unfortunately, diagnostic imaging did not provide a clear diagnosis which reinforces the importance of clinical sense. Conservative management was attempted with no success as the clinical status continued to worsen. The clinical decision was obvious facing an acute abdomen and surgery was crucial to the resolution of the case. A multidisciplinary approach is fundamental in management of these cases [17]. From the obstetrical point of view, MD contributed to poor outcomes, specifically cesarean section performance, iatrogenic preterm delivery, and newborn prematurity.

## 4. Conclusion

Although extremely rare, Meckel's diverticulitis is an important cause of acute abdomen during pregnancy. Its diagnosis implies a high index of clinical suspicion and must be considered especially when causes like appendicitis seem less plausible. Perforation of MD is more frequent in pregnant women. Symptomatic MD is associated with poor outcomes in pregnancy; therefore, a continuous monitoring, multidisciplinary approach, and an early and active management considering surgery are essential.

## Data Availability

The data used to support this study are available in this article.

## Supplementary Materials

Supplementary Materials (1) CARE guidelines with corresponding lines on the manuscript. (2) Original patient informed consent as it was different from Hindawi's consent (“if they use another form, they must provide a blank copy of the consent form on submission”). (3) Signed patient informed consent.
