# Supplementary material for: Meckel's Diverticulum: The “Great Mimic” but Often a Forgotten Cause of Acute Abdomen during Pregnancy
Source: Case Rep Obstet Gynecol. 2022 Jun 7;2022:2383075. doi: 10.1155/2022/2383075 (PMC9197602; doi:10.1155/2022/2383075)
Supplement: Supplementary Materials — (1) CARE guidelines with corresponding lines on the manuscript. (2) Original patient informed consent as it was different from Hindawi's consent (“if they use another form, they must provide a blank copy of the consent form on submission”). (3) Signed patient informed consent. [file 2383075.f1.pdf]

CARE case report guidelines

CARE Checklist of information to include when writing a case report

(cc) BY-NC-ND

Topic	Item	Checklist item description	Reported on Line
Title	1	The diagnosis or intervention of primary focus followed by the words "case report"	1
Key Words	2	2 to 5 key words that identify diagnoses or interventions in this case report, including "case report"	10
Abstract (no references)	3a	Introduction: What is unique about this case and what does it add to the scientific literature?	13, 14
	3b	Main symptoms and/or important clinical findings	15, 16
	3c	The main diagnoses, therapeutic interventions, and outcomes	17, 18
	3d	Conclusion—What is the main "take-away" lesson(s) from this case?	1, 2
Introduction	4	One or two paragraphs summarizing why this case is unique (**may include references**)	25, 26
Patient Information	5a	De-identified patient specific information.	29
	5b	Primary concerns and symptoms of the patient.	30, 31
	5c	Medical, family, and psycho-social history including relevant genetic information	32, 33
	5d	Relevant past interventions with outcomes	-
Clinical Findings	6	Describe significant physical examination (PE) and important clinical findings.	35-37
Timeline	7	Historical and current information from this episode of care organized as a timeline	-
Diagnostic Assessment	8a	Diagnostic testing (such as PE, laboratory testing, imaging, surveys).	37-41, 46
	8b	Diagnostic challenges (such as access to testing, financial, or cultural)	82-86, 100
	8c	Diagnosis (including other diagnoses considered)	48-50
	8d	Prognosis (such as staging in oncology) where applicable	-
Therapeutic Intervention	9a	Types of therapeutic intervention (such as pharmacologic, surgical, preventive, self-care)	43, 52, 53
	9b	Administration of therapeutic intervention (such as dosage, strength, duration)	-
	9c	Changes in therapeutic intervention (with rationale)	52, 53
Follow-up and Outcomes	10a	Clinician and patient-assessed outcomes (if available)	53, 54, 60
	10b	Important follow-up diagnostic and other test results	59
	10c	Intervention adherence and tolerability (How was this assessed?)	-
	10d	Adverse and unanticipated events	52
Discussion	11a	A scientific discussion of the strengths AND limitations associated with this case report	67,68, 141, 142
	11b	Discussion of the relevant medical literature **with references**.	67,68
	11c	The scientific rationale for any conclusions (including assessment of possible causes)	145-151
	11d	The primary "take-away" lessons of this case report (without references) in a one paragraph conclusion	156-161
Patient Perspective	12	The patient should share their perspective in one to two paragraphs on the treatment(s) they received	-
Informed Consent	13	Did the patient give informed consent? Please provide if requested	Yes ☒ No ☐

PATIENT INFORMED CONSENT

I ____, give my consent for the clinic information/photographs about myself relating to my disease/medical condition to appear in the journal, associated publications or translations or those to whom the journal licences its content. I have not seen the material to be submitted to the journal.

I understand the following:

(1). Use of this consent form does not waive your right to privacy. The Information will be published without your name attached. You should understand, however, that complete anonymity cannot be guaranteed. It is possible that somebody somewhere, for example somebody who looked after you when in hospital or a relative, may identify you.

(2). The text of the article will be edited for style, grammar, consistency, and length.

(3). The Information may be published in the journal, which is online and freely accessible worldwide to both health professionals, journalists, the public and others. The material may subsequently be used in other presentations at seminars or conferences, etc.

(4). You can revoke your consent at any time before publication, but once the Information has been committed to publication it will not be possible to revoke the consent.

(5). Patient and their family should note, they cannot normally expect to derive any financial benefit from the publication of the case.

(6). An original of the signed form will be held at the treating institution and the authors may be required to prove that consent was obtained.

(7). The author attests that an informed consent process was followed when this form was completed, with the patient sufficient time to consider its implications.

(8). This consent must be signed in English and Portuguese.

Name (patient): ____

Signed (patient): ____ Date: ____________

Name (author): ____

Signed (author): ____ Date: ____________

PATIENT INFORMED CONSENT

I Daria Migotina Rodrigues, give my consent for the clinic information/photographs about myself relating to my disease/medical condition to appear in the journal, associated publications or translations or those to whom the journal licences its content. I have not seen the material to be submitted to the journal.

I understand the following:

(1). Use of this consent form does not waive your right to privacy. The Information will be published without your name attached. You should understand, however, that complete anonymity cannot be guaranteed. It is possible that somebody somewhere, for example somebody who looked after you when in hospital or a relative, may identify you.

(2). The text of the article will be edited for style, grammar, consistency, and length.

(3). The Information may be published in the journal, which is online and freely accessible worldwide to both health professionals, journalists, the public and others. The material may subsequently be used in other presentations at seminars or conferences, etc.

(4). You can revoke your consent at any time before publication, but once the Information has been committed to publication it will not be possible to revoke the consent.

(5). Patient and their family should note, they cannot normally expect to derive any financial benefit from the publication of the case.

(6). An original of the signed form will be held at the treating institution and the authors may be required to prove that consent was obtained.

(7). The author attests that an informed consent process was followed when this form was completed, with the patient sufficient time to consider its implications.

(8). This consent must be signed in English and Portuguese.

Name (patient): Daria Migotina Rodrigues

Signed (patient): [signature] Date: 23.03.2021

Name (author): Beatriz Dias Cordeiro Féria

Signed (author): [signature] Date: 23/3/21
